# Extracellular vesicles from GABAergic but not glutamatergic neurons protect against neurological dysfunction following cranial irradiation

**DOI:** 10.1038/s41598-024-62691-y

**Published:** 2024-05-28

**Authors:** Sarah M. Smith, Kashvi Ranjan, Brianna M. Hoover, Olivia G. G. Drayson, Munjal M. Acharya, Eniko A. Kramár, Janet E. Baulch, Charles L. Limoli

**Affiliations:** 1 Department of Radiation Oncology, University of California Irvine, Medical Sciences I, Room B-146B, Irvine, CA 92697-2695 USA; 2 Department of Anatomy and Neurobiology, University of California, Irvine, CA USA; 3 Department of Neurobiology and Behavior, University of California, Irvine, CA USA

**Keywords:** Neuroscience, Stem cells in the nervous system

## Abstract

Cranial irradiation used to control brain malignancies invariably leads to progressive and debilitating declines in cognition. Clinical efforts implementing hippocampal avoidance and NMDAR antagonism, have sought to minimize dose to radiosensitive neurogenic regions while normalizing excitatory/inhibitory (E/I) tone. Results of these trials have yielded only marginal benefits to cognition, prompting current studies to evaluate the potential of systemic extracellular vesicle (EV) therapy to restore neurocognitive functionality in the irradiated brain. Here we tested the hypothesis that EVs derived from inhibitory but not excitatory neuronal cultures would prove beneficial to cognition and associated pathology. Rats subjected to a clinically relevant, fractionated cranial irradiation paradigm were given multiple injections of either GABAergic- or glutamatergic-derived EV and subjected to behavioral testing. Rats treated with GABAergic but not glutamatergic EVs showed significant improvements on hippocampal- and cortical-dependent behavioral tasks. While each treatment enhanced levels of the neurotrophic factors BDNF and GDNF, only GABAergic EVs preserved granule cell neuron dendritic spine density. Additional studies conducted with GABAergic EVs, confirmed significant benefits on amygdala-dependent behavior and modest changes in synaptic plasticity as measured by long-term potentiation. These data point to a potentially more efficacious approach for resolving radiation-induced neurological deficits, possibly through a mechanism able to restore homeostatic E/I balance.

## Introduction

With ever increasing improvements in the diagnosis and treatment of cancer, survivorship continues to rise, however, survival is accompanied by a compromised quality of life^1^. Cranial irradiation used to treat primary and secondary malignancies of the brain may be an effective cancer therapy, still it results in long-term neurocognitive side effects, including progressive and frequently debilitating decrements in learning, memory, and mood that do not resolve over time^2–4^. To date, effective long-term solutions to this unmet medical need remain elusive. Much of our past work has attempted to address this problem though transplantation approaches designed to increase the number of neural stem and progenitor cells depleted after cranial radiotherapy. Rodents subjected to whole brain irradiation exhibit significant cognitive impairments spanning multiple brain regions, deficits that in large part could be ameliorated by intrahippocampal human stem cell transplantation^5–7^. Follow-up studies advanced the translational applicability of these approaches by substituting stem cells with stem cell derived extracellular vesicles (EVs)^8^. Interestingly, almost identical functional benefits were obtained with EV transplantation in the irradiated brain, thereby obviating the need for invasive surgeries and minimizing the risks of teratoma formation and immunorejection.

EV is a term used to describe a variety of nanometer-scale membrane-bound vesicles that are secreted by cells and that can participate in paracrine and endocrine signaling^9–11^. Contents of EV include lipids, nucleic acids including genomic DNA, microRNAs (miRNAs), and mRNAs, proteins, and in some cases mitochondria^11^. Based on the evidence available to date, miRNAs are considered critical functional cargo within EV given that a single miRNA can impact multiple gene targets and signaling pathways^12^. EV are considered specialized long distance mediators of intercellular communication and the small size and lipid-heavy composition of the particles facilitates their translocation across the blood–brain barrier^13,14^, ideally enabling them to deliver their bioactive cargo into target cell populations in the brain. The substitution of EV for stem cells to resolve neurocognitive sequelae has the distinct advantages of eliminating the risk of teratoma formation, minimizing complications associated with immunogenicity^15^ and providing systemic route of administration that negates the need for invasive surgical procedures. In these studies, the EV treatments restored balance in pre- and post-synaptic protein levels, neuronal signaling and reduced the persistent neuroinflammation found within the irradiated brain. Follow up work documented the ability of injected EV to migrate extensively throughout the irradiated brain^16^ and deliver beneficial bioactive cargo upon fusing with target cells, in particular the miRNA miR-124^17^.

To further our understanding of the beneficial effects of EVs we now evaluate the functional consequences of EV administration in the irradiated rat brain using EVs from two distinctly different neuronal cell types. In this study, we test the hypothesis that EV isolated from human iPSC-derived GABAergic neuronal cultures would resolve adverse radiation-induced side effects in contrast to EV isolated from human iPSC-derived glutamatergic neuronal cultures. The rationale for these experiments is based upon observations that changes in excitatory/inhibitory balance may contribute to network-level disruptions and the development of neurodegenerative disorders and cognitive decline^18^. Our data from behavioral experiments demonstrate improved behavioral performance on learning and memory tasks and neuron architectural protection in increased spine density in the hippocampus of the GABAergic but not glutamatergic EV-treated irradiated brain. Interestingly, though, the levels of neurotrophic factors were affected similarly by both types of EV.

## Results

**Figure 1:**
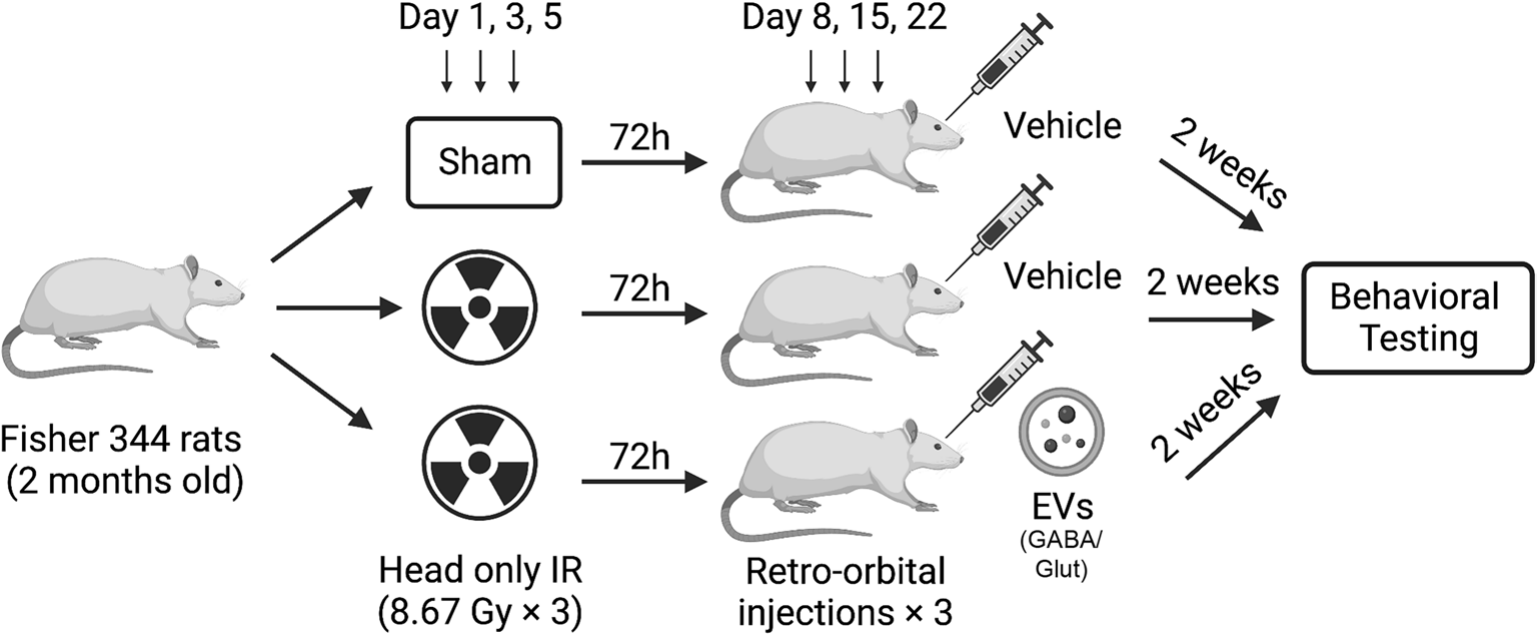
Experimental design for irradiation and retro-orbital (RO) injection ± GABAergic or glutamatergic neuron derived extracellular vesicles (EVs).

An experimental timeline is presented to clarify the cohorts used in this study (Fig. 1). For each GABAergic and the glutamatergic EV studies, rats were divided into three experimental groups (*N* = 16 animals per group): a control group that received sham irradiation (exposure to anesthesia) and vehicle (hibernation buffer) retro-orbital (RO) injections, a group that received fractionated irradiation and vehicle injections, and a group that received fractionated irradiation and injections of GABAergic EVs. A second, independent cohort of rats were grouped and treated similarly but received injections of glutamatergic neuron derived EVs. Irradiated experimental groups received three fractionated doses of whole-brain irradiation of 8.67 Gy × 3 (total 26 Gy) over the course of 5 days (i.e. on days 1, 3, and 5).

### Extracellular vesicle characterization and tracking

**Figure 2:**
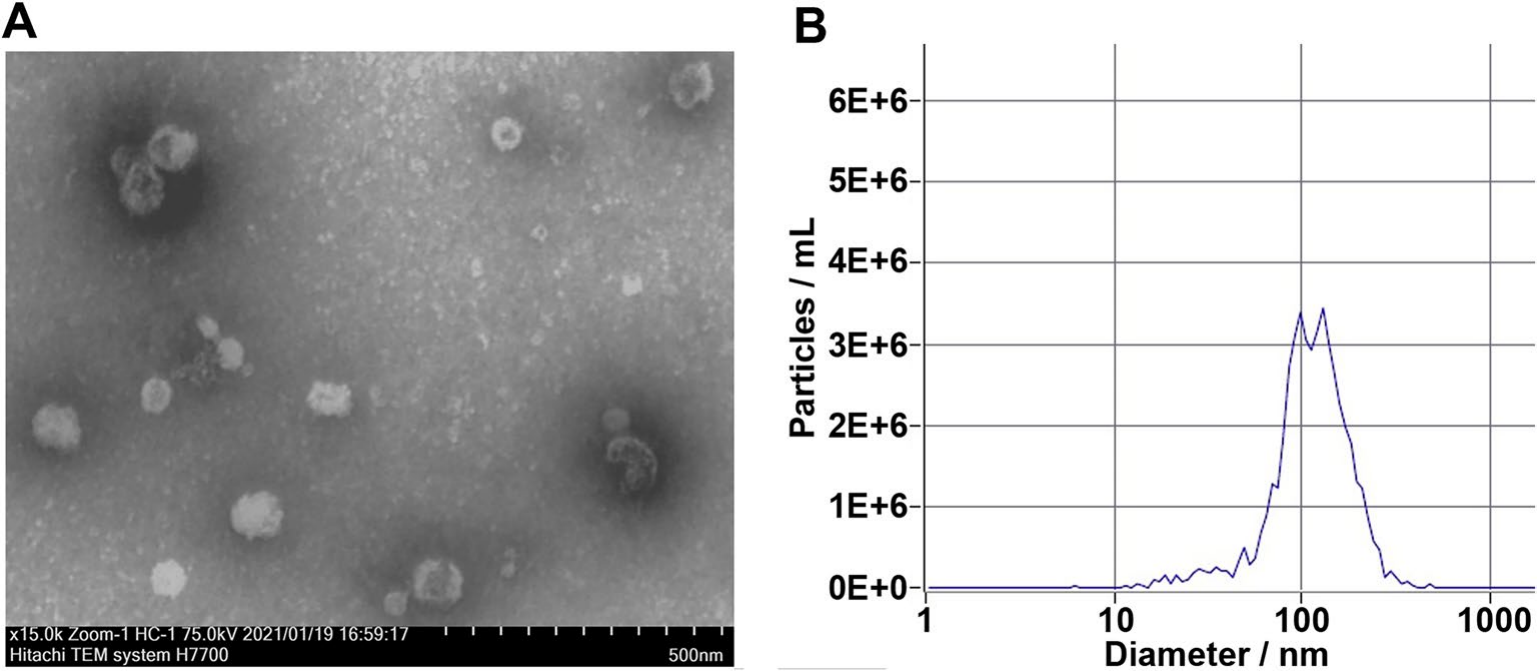
Characterization of extracellular vesicles (EVs). (**A**) Representative electron microscopy image depicts typical EV morphology and size. (**B**) Representative graph from ZetaView analysis showing the average size distribution of the EVs.

**Figure 3:**
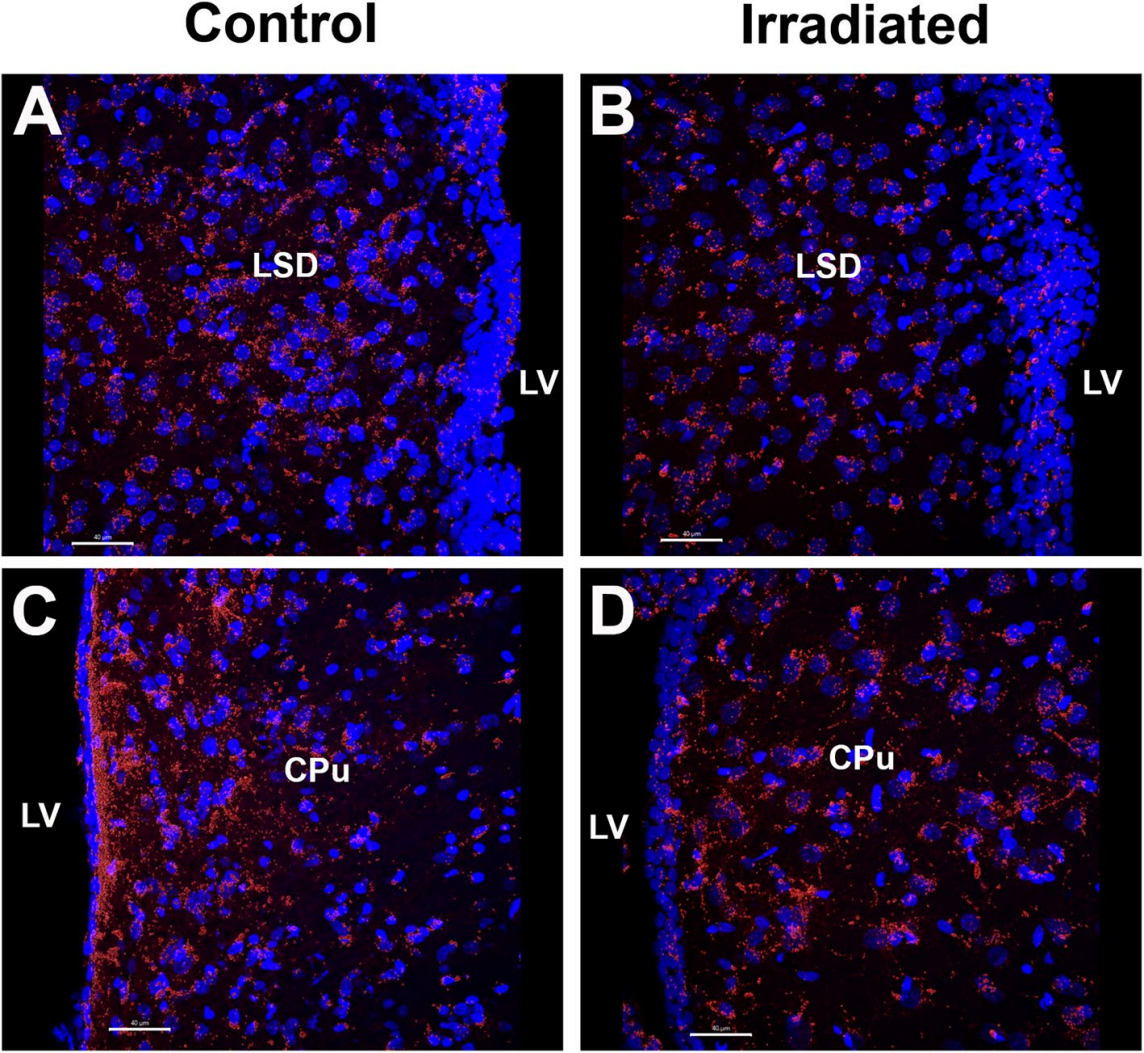
In vivo tracking of extracellular vesicles (EVs). Confocal Z-stacks collected at × 40 magnification qualitatively demonstrate that retro-orbitally injected EVs migrated through the lateral ventricle (LV) in the lateral septal nucleus (LSD, (**A**,**B**) and caudate putamen (CPu, **C**,**D**) subregions of control (**A**,**C**) and irradiated (**B**,**D**) rats. EV red; DAPI stained nuclei, blue; Scale bars: 40 µm.

The morphology of EVs was analyzed by electron microscopy and revealed intact vesicles of a relatively uniform size (Fig. 2A). Further characterization was conducted to quantify the yield of size distributions of EVs and showed that the majority of EVs isolated were within the mean range of 100 nm in diameter (Fig. 2B). Control studies were then performed to validate penetrance into the brain (Fig. 3).

To interrogate the distribution of EVs administered by RO injections throughout the hippocampus, GABAergic neuron derived EVs were labeled with PKH26 prior to injection. Animals were divided into two groups (*N* = 2 animals per group): a control group that received sham irradiation (exposure to anesthesia; Fig. 3A,C) and an irradiated group that received 26 Gy total fractionated irradiation (8.67 Gy × 3; Fig. 3B,D). At 72 h post-irradiation, the animals received injections of PKH26-labeled EVs in 100 μm hibernation buffer. Confocal microscopy revealed the presence and migration of the labeled EVs through the indicated subregions of the brain at 24 h post-RO injection (Fig. 3).

### Extracellular vesicles from GABAergic but not glutamatergic neurons rescue performance on behavioral tasks following irradiation

**Figure 4:**
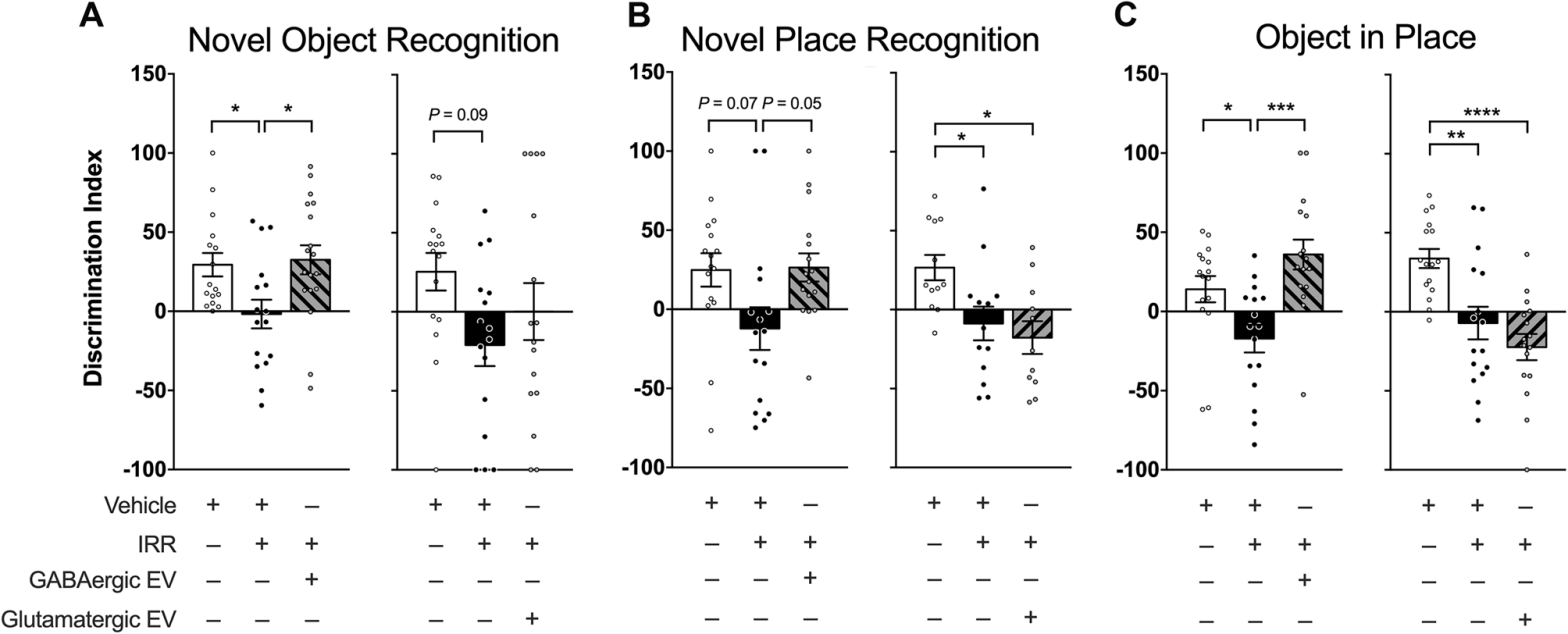
Treatment with GABAergic extracellular vesicles (EVs) improves cognitive performance. (**A**) Novel Object, (**B**) Novel Place, and (**C**) Object in Place Recognition performance following irradiation. Animals were treated with GABAergic or glutamatergic neuron derived EVs following fractionated irradiation (left and right panels, respectively). Radiation-induced decrements in performance on these tasks are not mitigated by glutamatergic EVs. Data are presented as the mean ± SEM (*N* = 16 animals/group). *P < 0.05, **P < 0.01, ***P < 0.001, ****P < 0.0001; one-way ANOVA with Bonferroni’s multiple comparisons test.

The Novel Object Recognition (NOR) test assesses episodic memory retention that is dependent on the frontal and pre-frontal cortex and is known to be impaired following irradiation^6,8^. Concurrent with other spontaneous exploration tasks, rats with normal cognitive function exhibit a preference for novel objects. Animals treated with vehicle alone were able to discriminate between the novel and familiar objects and exhibited a preference for the novel objects as expected for cognitively intact animals (mean discrimination index = 29.45%), while irradiated (IRR) animals showed no ability to discriminate between novel and familiar objects (mean discrimination index = − 1.72%; Fig. 4A). Significant overall group effects were found (F_(2, 47)_ = 4.738; *P* = 0.0134) and the differences in performance between the control group and the IRR group were statistically significant (F_(2, 47)_ = 4.738; *P* = 0.0480). Irradiated animals treated with GABAergic EVs demonstrated significant improvement of cognitive function (mean discrimination index = 32.55%) compared to animals that received IRR alone (F_(2, 47)_ = 4.738; *P* = 0.0206; 4A, left panel). In contrast, the mean discrimination indices of the control group and the GABAergic EV group were statistically indistinguishable (F_(2, 47)_ = 4.738; *P* > 0.9999; Fig. 4A, left panel).

Irradiated animals in the glutamatergic EV cohort did not exhibit improved cognitive performance in the NOR test. In this cohort, there was no significant overall group effect (F_(2, 45)_ = 2.516; *P* = 0.0921). Performance in the control group (mean discrimination index = 25.39%) was greater than that of the IRR group (mean discrimination index = − 21.00%), but this difference was not statistically significant (F_(2, 45)_ = 2.516; *P* = 0.0921; Fig. 4A, right panel). Glutamatergic EV-treated animals likewise failed to discriminate between novel and familiar (mean discrimination index = 0.1461%), and their performance was statistically comparable to that of the IRR rats (F_(2, 45)_ = 2.516; *P* = 0.9377; Fig. 4A, right panel). These data show that administration of GABAergic EVs significantly improves the cognitive ability of animals to distinguish between novel and familiar objects (Fig. 4A). However, administration of glutamatergic EVs fails to improve cognition in NOR following irradiation.

Next, the Novel Place Recognition (NPR) test was conducted to evaluate the functionality of the hippocampus and the medial prefrontal cortex (mPFC)^19,20^ following IRR and treatment with EVs (Fig. 4B). The NPR test detected an overall group effect among animals in the GABAergic cohorts (F_(2, 45)_ = 3.829; *P* = 0.0291; Fig. 4B, left panel). Vehicle-treated and GABAergic EV-treated irradiated animals had higher mean discrimination indices (24.88% and 26.45%, respectively) than the IRR group (mean discrimination index = − 12.17%); however, no statistical significance was observed between control and IRR animals (F_(2, 45)_ = 3.829; *P* = 0.0705) nor between the IRR and EV-treated animals (F_(2, 45)_ = 3.829; *P* = 0.0554; Fig. 4B, left panel).

Significant overall group effects were demonstrated among the glutamatergic EV cohorts with the NPR task (F_(2, 33)_ = 5.602; *P* = 0.0080; Fig. 4B, right panel). Vehicle-treated animals showed statistically superior performance (mean discrimination index = 26.60%; F_(2, 33)_ = 5.602; *P* = 0.0415) compared to IRR animals (mean discrimination index = − 8.788%). However, there was no statistical difference (F_(2, 33)_ = 5.602; *P* > 0.9999) in performance between the IRR group and the glutamatergic EV-treated irradiated group (mean discrimination index = − 17.73%, Fig. 4B; right panel). There was a statistically significant difference in mean discrimination indices between control animals and glutamatergic EV-treated irradiated animals (F_(2, 33)_ = 5.602; *P* = 0.0111, Fig. 4B; right panel). These data show that treatment with GABAergic EVs improves irradiation-induced detriments in NPR performance, though not significantly, while glutamatergic EVs fail to impart any benefits.

Next, the Object in Place (OiP) task was performed to interrogate spatial memory retention that is facilitated by the hippocampus and pre-frontal cortex^19,20^. Significant group effects were observed among GABAergic EV cohorts (F_(2, 45)_ = 9.104; *P* = 0.0005; Fig. 4C, left panel), and there were statistically significant increases in the mean discrimination indices compared to IRR for both the control group (F_(2, 45)_ = 9.104; *P* = 0.0492) and the GABAergic EV-treated group (F_(2, 45)_ = 9.104; *P* = 0.0003). As expected, irradiated animals were unable to discriminate between novelty and familiarity (mean discrimination index = − 17.13%; Fig. 4C, left panel). The performances of the control group (mean discrimination index = 14.05%) and the GABAergic EV-treated irradiated group (mean discrimination index = 35.95%) were statistically similar (F_(2, 45)_ = 9.104; *P* = 0.2599; Fig. 4C, left panel).

For OiP performance in glutamatergic EV cohorts, significant group effects were observed (F_(2, 32)_ = 7.226; *P* = 0.0054, Fig. 4C, right panel). The mean discrimination index of the IRR group (mean discrimination index = − 1.263%) was significantly reduced (F_(2, 32)_ = 7.226; *P* = 0.0044) compared to the control group (mean discrimination index = 40.71%; Fig. 4C, right panel). In contrast, the difference between the mean discrimination index of the irradiated group and the EV-treated group did not reach significance (F_(2, 32)_ = 7.226; *P* = 0.6251), and both groups failed to exhibit a preference for novelty (Fig. 4C, right panel). These data show that radiation exposure eliminates the capacity to identify novelty on the OiP task, and this deleterious effect is reversed with treatment of GABAergic but not glutamatergic EVs.

Additional studies focused on GABAergic EV to evaluate their potential impact on anxiety related disorders using subsets of the behavioral cohorts. Although not statistically significant, irradiated cohorts treated with GABAergic EV did exhibit a trend toward increased transitions between light and dark regions compared to untreated irradiated cohorts (Supplementary Fig. 1).

### Extracellular vesicles rescue neurotrophin levels following irradiation

**Figure 5:**
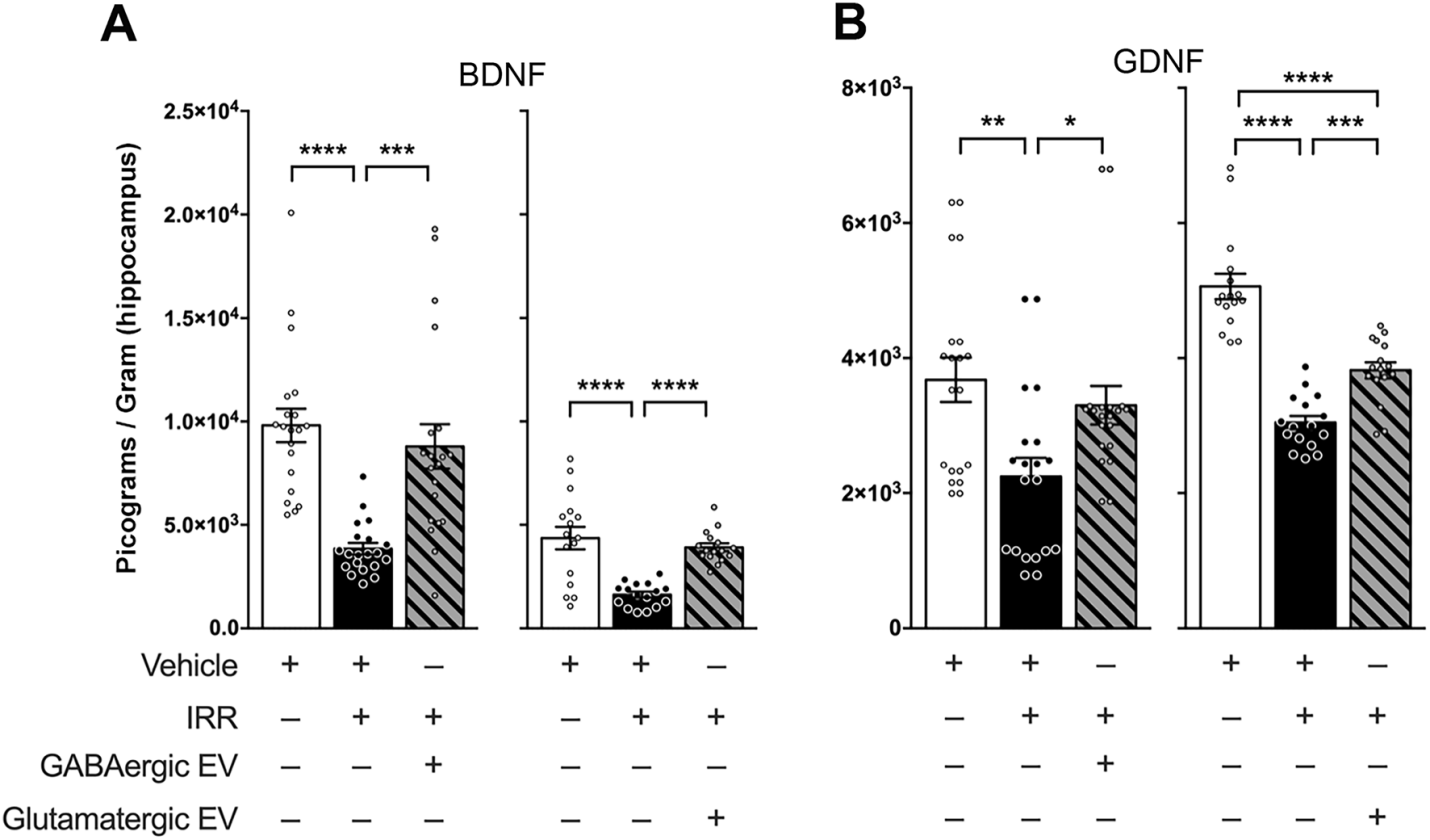
Radiation-induced reduction in neurotrophic factors is ameliorated by GABAergic and glutamatergic extracellular vesicles (EVs). (**A**,**B)** Levels of brain-derived neurotrophic factor (BDNF) are significantly decreased following irradiation (IRR) compared to controls. Treatment with (**A**) GABAergic (left panel) and glutamatergic (right panel) neuron derived EVs rescued BDNF levels to near that of controls. (**B**) Levels of glial cell-derived neurotrophic factor (GDNF) are significantly reduced upon IRR and treatment with GABAergic (left panel) and glutamatergic (right panel) neuron derived EVs significantly increase GDNF. Data are presented as the mean ± SEM (*N* = 4–5 animals/group). *P < 0.05; **P < 0.01, ***P < 0.001, ****P < 0.0001; one-way ANOVA with Bonferroni’s multiple comparisons test.

To determine whether neurotrophins could be responsible for the differential cognitive performance of animals receiving GABA and glutamatergic extracellular vesicles, neurotrophin levels in the hippocampus were quantified by ELISA in a randomly selected subset of the behaviorally tested animals (Fig. 5). Analysis of brain-derived neurotrophic factor (BDNF) showed significant overall group effects among GABAergic cohorts (F_(2, 57)_ = 16.17; *P* < 0.0001; Fig. 5A, left panel). BDNF was significantly decreased following irradiation compared to controls (F_(2, 57)_ = 16.17; *P* < 0.0001), and treatment with GABAergic EV following irradiation significantly increased BDNF (F_(2, 57)_ = 16.17; *P* = 0.0001). BDNF was completely rescued with GABAergic EVs, as BDNF levels between controls and the irradiated GABAergic EV group were similar (F_(2, 57)_ = 16.17; *P* > 0.9999).

Analysis of BDNF among the glutamatergic cohort treatments showed significant overall group effects (F_(2, 45)_ = 18.30; *P* < 0.0001). As expected, irradiated animals had significantly decreased BDNF compared to controls (F_(2, 45)_ = 18.30; *P* < 0.0001; Fig. 5A, right panel). Interestingly, glutamatergic EV-treated animals exhibited significantly increased BDNF levels in the hippocampus over that of the irradiated group (F_(2, 45)_ = 18.30; *P* < 0.0001), with BDNF levels statistically similar to that of the control group (F_(2, 45)_ = 18.30; *P* > 0.9999).

Upon analysis of glial cell line derived neurotrophic factor (GDNF), significant group effects (F_(2, 57)_ = 6.186; *P* = 0.0037) were observed among GABAergic groups (Fig. 5B). There was a statistically significant reduction in GDNF upon irradiation (F_(2, 57)_ = 6.186; *P* = 0.0038) relative to controls (Fig. 5B, left panel). This reduction in GDNF was reversed with treatment of GABAergic EVs, which caused a statistically significant increase in GDNF compared to irradiated animals (F_(2, 57)_ = 6.186; *P* = 0.0459), and GDNF between controls and EV-treated irradiated animals were statistically equivalent (F_(2, 57)_ = 6.186; *P* > 0.9999).

Likewise, GDNF levels among glutamatergic cohorts yielded significant overall group effects (F_(2, 45)_ = 52.57; *P* < 0.0001; Fig. 5A, right panel). GDNF was significantly decreased in irradiated animals compared to controls (F_(2, 45)_ = 52.57; *P* < 0.0001); and glutamatergic EV-treated animals exhibited significantly higher GDNF relative to the irradiated group (F_(2, 45)_ = 52.57; *P* = 0.0010). However, there was also a statistically significant difference in GDNF levels between the control rats and the glutamatergic EV-treated irradiated animals (F_(2, 45)_ = 52.57; *P* < 0.0001; Fig. 5A, right panel). Collectively, these data demonstrate that the neutrotrophic factors BDNF and GDNF are significantly reduced in the hippocampus following irradiation. While treatment with both GABAergic and glutamatergic neuron derived EVs rescue levels of BDNF, GDNF is mostly attenuated by treatment of GABAergic extracellular vesicles and partially attenuated by treatment of glutamatergic EVs.

### GABAergic extracellular vesicles mitigate radiation-induced decline in dendritic spine density

**Figure 6:**
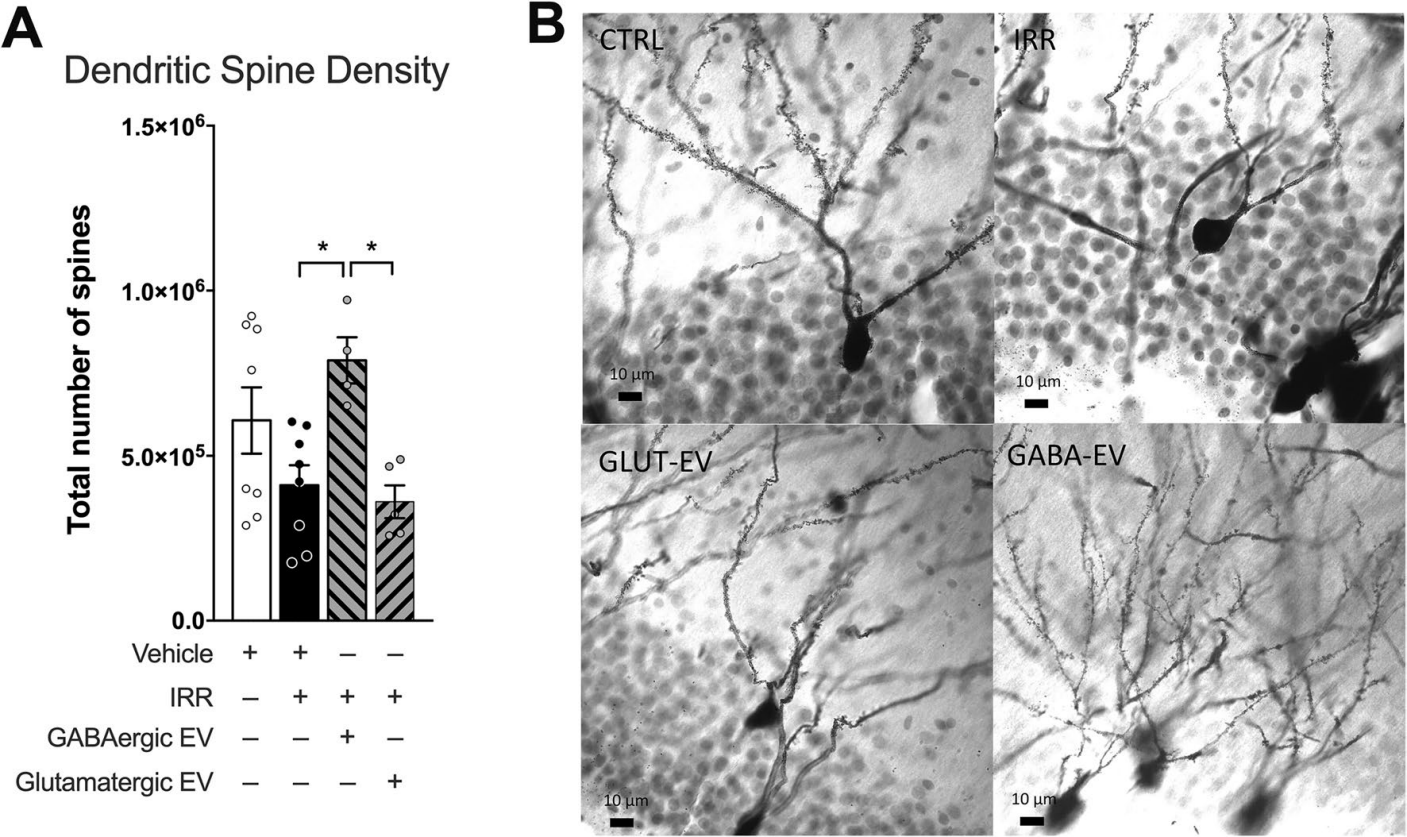
Dendritic spine density in the dentate gyrus is diminished following irradiation and is rescued with GABAergic but not glutamatergic extracellular vesicles. (**A**) Data represent total spine density counts along dendritic arbors across a constant region of interest in the hippocampus. Data are presented as the mean ± SEM (*N* = 4 animals/group). *P < 0.05, one-way ANOVA with Bonferroni's multiple comparisons test. (**B**) Representative images. Scale bars: 10 µm.

Morphologic alterations of the dentate gyrus were assessed by quantification of dendritic spine density (Fig. 6). This analysis demonstrated significant overall group effects (F_(3, 21)_ = 4.565; *P* = 0.0130; Fig. 6). There was a reduction in dendritic spine density in the irradiated as compared to the control group, though this difference was not statistically significant (F_(3, 21)_ = 4.565; *P* = 0.2516). However, treatment of the irradiated group with GABAergic neuron derived EVs was able to mitigate this decline, with the GABAergic EV-treated group showing a significant increase in spine density (F_(3, 21)_ = 4.565; *P* = 0.0304) over that of the irradiated group; Fig. 6). The application of glutamatergic EVs, however, did not rescue dendritic spine density following irradiation and spine densities between GABAergic and glutamatergic EV-treated animals were significantly different (F_(3, 21)_ = 4.565; *P* = 0.0243).

### GABAergic extracellular vesicles partially mitigate the inhibition of long-term potentiation after irradiation

To explore further whether changes in dendritic spine density might compromise neurotransmission, additional studies were conducted with GABAergic EV to evaluate their potential impact on long-term potentiation (LTP), an electrophysiological measure of synaptic plasticity^21^. Data showed that irradiated cohorts exhibited a significant drop in mean potentiation that was partially restored by GABAergic EV treatments but that did not reach statistical significance (Supplementary Fig. 2).

## Discussion

Our previous work in mice demonstrated cranial irradiation-induced cognitive impairments with no lessening of severity as long as 6 months post-exposure that could be ameliorated with intracranial or retro-orbitally injected stem cell derived EVs^17^. The aim of the current study was to evaluate the functional consequences of administering EVs from two distinct neuronal origins to test the hypothesis that including iPSC-derived GABAergic neuron-derived EVs help resolve adverse radiation-induced sequelae in contrast to EVs isolated from iPSC-derived glutamatergic neurons. Our rationale followed cross-disciplinary evidence pointing to changes in the excitatory/inhibitory (E/I) balance hypothesized to be contributory, if not causal, to the network-level disruptions and the development of neurodegenerative disorders and cognitive decline^18^. Certain proteinopathies can also trigger excessive astrocytic glutamate release which can be attenuated by NMDAR antagonism^22^.

Brain tumor patients are routinely treated with radiation, used as an effective means for forestalling primary tumor and secondary metastatic progression^23,24^. Neurocognitive decline in brain tumor survivors has been recognized as the second most important measure of treatment success following tumor cure^1,25^. This unmet medical need has ushered in hippocampal avoidance treatment planning in efforts to spare radiosensitive neurogenic regions and reduce treatment-induced deficits in cognition^1,2^. Importantly, the uncompetitive NMDAR antagonist memantine approved to treat Alzheimer’s disease^26^, is now part of standard of care radiotherapy used in conjunction with hippocampal avoidance plans to help minimize the adverse effects of radiation exposure on cognition^25^. Interestingly, a related drug nitrosynapsin could also prove beneficial in this setting. Nitrosynapsin targets extrasynaptic NMDAR to attenuate tonic glutamatergic currents and ameliorate synaptic loss^22,27^. Based on the foregoing, and the role of inhibitory neurons to regulate a largely excitatory organ, we posited that EV secreted from inhibitory versus excitatory neuronal cultures could mediate beneficial paracrine signaling in the irradiated brain.

Rats subjected to fractionated cranial irradiation targeted to match the biologically effective dose (BED ~ 100, assuming an α/β ratio of 3 for the normal brain) used for the treatment of glioblastoma patients were given repeated systemic injections of GABAergic or glutamatergic EV prior to cognitive testing. In nearly every instance, radiation-induced decrements were ameliorated by GABAergic but not glutamatergic EV. Interestingly, whole brain acute 11 Gy irradiation has been shown to reduce inhibitory neurotransmitters, including GABA and glycine, GABAa receptors, and increase excitatory neurotransmitters, including glutamate^28^. High dose gamma irradiation using a 60 Gy dose selectively reduced GABAa receptor-mediated inhibitory neurotransmission^29^. Taken together, these reports indicate neurochemical and neurotransmission imbalances in the irradiated brain. Past work in the neurodegenerative literature has substantiated that augmenting BDNF and/or GDNF through stem cell transplantation could resolve memory impairments and synaptic density^30–33^, providing some of the rationale for evaluating the potential role for these factors in the present study. While the benefits in cognition were noteworthy, these changes did not track with enhanced levels of the neurotrophic factors BDNF or GDNF, as both EV sources increased the levels of each neurotrophic factor to quantitatively similar levels. Interestingly, GABAergic but not glutamatergic EV preserved and/or increased dendritic spine density in the hippocampal dentate gyrus, which may underlie the trend toward restoration of LTP that was inhibited after irradiation. Since the spatial exploration tasks used interrogate hippocampal function, a preservation of spine density and a modest attenuation of LTP in the dentate gyrus provides one plausible explanation for the beneficial effects of GABAergic but not glutamatergic EV.

Much of our past work has documented the many benefits of stem cell transplantation and more recently stem-cell derived EV in preserving functionality of the irradiated rodent brain^6,7,16,34^. Initial work implementing intrahippocampal transplantation of various human neural stem cells indicated the neurocognitive benefits of such approaches that increased expression of the activity-regulated cytoskeleton-associated protein (Arc) density in the brain following cranial irradiation^7,35^. The ability to ameliorate radiation-induced cognitive dysfunction and associated pathology was demonstrated using transplanted and systemically injected stem-cell derived EVs into athymic nude rats^8^ and subsequently in wild type mice^17^, thereby obviating the need for surgery and immune-suppression. In that latter work, analysis of the bioactive cargo isolated from the EV, identified miR-124 as a beneficial component of that cargo^17^.

Together, our work has consistently demonstrated that EVs are exceedingly effective paracrine mediators over extended regions of the brain^8,17,36^. One past study demonstrated migration of cranially injected EV from one hemisphere to the other (ipisilateral to contralateral), where similar numbers of EV were found within each hippocampus^16^. Our more recent work utilizing retro-orbital injection of EVs demonstrated their capability to readily cross the blood–brain barrier, reaching critical brain regions including the hippocampus^17,36^. Although we only tracked GABAergic EVs to the lateral ventricle in the lateral septal nucleus and caudate putamen subregions of the control and irradiated brain, this past evidence suggests a certain level of penetrance of both types of EVs to the hippocampal subfields. While we do not have direct evidence confirming this assertion, or evidence as to the specific cell types targeted by the EV, the beneficial functional effects of GABAergic EV do support the widespread trophic benefits of this approach.

While the current work did not analyze the membrane bound and internal constituents of the GABAergic and glutamatergic EVs, the probable differences between miRNA, synaptic components and/or neurotransmitter cargo previously characterized in human embryonic stem cell derived EV^37^ are likely to have influenced our neurological outcomes. The differential distribution of synaptic and non-synaptic GABAergic receptors isolated from synaptosomes both in vivo and from cultured neurons suggest, that the EVs isolated in this study may be enriched in GABAergic receptors distributed at the neuronal surface and associated with lipid rafts^38^. This may promote beneficial GABAergic signaling to normalize the excitatory/inhibitory balance in the irradiated brain. The use of stem cell derived EV to treat various neurological orders has been reviewed^9–11^, and a recent study isolating EV from hippocampal interstitial fluid found the microglial EV proteome sensitive to sex, age, and Alzheimer’s pathology, opening the door to using brain derived EV as biomarkers to diagnose and stage neurodegenerative disease progression^39^. Further, cargo analysis and omics level studies will be required to provide more precise mechanistic context for our findings.

It is important to note that in our past work, and the work of others, we have not included stem cell or EV injected control mice for the simple reason that other rodent studies have clearly demonstrated that the intact normal brain was not affected by engraftment^7,40–42^. In each of those cases the stem cell or EV injected control mice exhibited functional or molecular measurements that were not statistically different from that of untreated control mice. Further, given the goal of translating our EV-based approach to clinical treatment, there is no rationale for including a cohort of tumor free control animals that have been EV injected.

Collectively, our work suggests that EV treatments have promise to lessen the adverse effects of cranial radiotherapy on cognitive function following clinical treatment of malignancies of the brain. These observations are not limited to just the adverse sequelae associated with brain cancers, as normal tissue injury following radiation therapy treatments of other cancers are also a common complication^43^. Indeed, a related study has demonstrated that EV injection of human neural stem cell derived EVs are capable of ameliorating lung fibrosis in mice following thoracic irradiation^37^. Addressing these persistent normal tissue injuries is a critically important issue for an ever-growing number of patients as cancer treatments become increasingly effective. However, a critical caveat to EV-based therapeutics is highlighted in our current study that demonstrates that all stem cell derived EVs are not created equally in their ability to ameliorate therapy effects and improve the quality of life for cancer survivors.

## Materials and methods

### Animals and irradiation

All animal procedures are in accordance with the NIH and approved by the University of California Institutional Animal Care and Use Committee and animal experiments follow ARRIVE guidelines and address the 10 essential criteria described therein. Eight-week-old male Fischer 344 rats (Crl:CD(Fischer 344), strain 002, Charles River Laboratories, Wilmington, MA) were maintained in standard housing conditions (20 °C ± 1 °C; 70% ± 10% humidity; 12 h:12 h light and dark cycle) and provided ad libitum access to food and water. Rats were anesthetized using an isoflurane gas system (VWR Mobile RC2; induction = 3.5% vol/vol isoflurane/oxygen) and radiation was delivered using a self-shielded 320 kV X-irradiator (X-RAD320 irradiator, Precision X-Ray, North Branford, CT) and lead shielding to facilitate head-only irradiation over a 2 cm^2^ area at a dose rate of 1 Gy/min. Control animals were anesthetized for the same amount of time as the irradiated animals to ensure corresponding isoflurane exposure, and handled identically to those undergoing irradiation including vehicle RO injections.

### Extracellular vesicles and isolation

GABAergic neuron-derived EVs were purified from conditioned culture media in which human induced pluripotent stem (iPS) cell-derived GABAergic neurons had been cultured (iCell GABANeurons, 01434, FUJIFILM Cellular Dynamics, Inc, Madison, WI). Similarly, glutamatergic neuron-derived EVs were purified from conditioned culture media in which iPS cell-derived glutamatergic neurons had been cultured (iCell GlutaNeurons, 01279, FUJIFILM Cellular Dynamics, Inc, Madison, WI). The fully differentiated, human GABAergic inhibitory neurons were comprised of > 95% cerebral cortical neurons possessing native electrical and biochemical activity and able to form neural networks and functional synapses. Similarly, the fully differentiated cortical neuronal cell lines used in this study while not purely homogeneous, were nonetheless ≥ 90% pure and able to form functional neural networks and synapses.

The purification and characterization of the EV followed previously published procedures^16,44^. Briefly, the conditioned culture medium was put through a 0.22-μm sterile filter as an initial purification before centrifugation at 300 × g for 10 min then at 100,000 × g for 90 min. EVs were then purified by ultracentrifugation in sterile Dulbecco’s phosphate-buffered saline at 100,000 × g for 120 min then collected and pooled in hibernation buffer.

EVs were analyzed by electron microscopy for morphology where the material was negatively stained by applying a drop of EV solution (final concentration approximately 1 mg/mL) directly onto a 300-mesh formvar-carbon-coated nickel grid (Electron Microscopy Sciences; Hatfield, PA), which was allowed to remain for approximately 60 s, after which excess solution was removed^45^. A drop of 1% aqueous uranyl acetate was then added onto the grid and allowed to remain for an additional 60 s, after which excess solution was removed and the grids allowed to dry. Material was imaged on a Hitachi 7500 transmission electron microscope equipped with an Advanced Imaging Technologies (AMT) digital camera. A digital point-to-point measuring tool was used to determine EV size distribution. Images were imported into Photoshop CS2 (Adobe Systems Inc.) where they were sized and optimized for contrast and brightness. The EVs were also characterized for size and number using a ZetaView PMX 110 particle analyzer (Particle Metrix GmbH; Meerbusch Germany).

### Extracellular vesicle labeling

For visualization and in vivo tracking, EVs were labeled with PKH26 (Sigma‐Aldrich, PKH26GL) prior to RO injection. The EVs were resuspended in Diluent C, incubated with Dye Solution for 4 min with intermittent mixing as per the manufacturer’s protocol. The dye was quenched with 1% bovine serum albumin in water, and the EVs were isolated through ultracentrifugation at 100,000 × g for 90 min and collected in hibernation buffer. For each animal, 1.5 × 10^10^ EVs were dyed and prepared for injection. Animals were irradiated or sham irradiated as described above, injected with EVs 48 h later and euthanized at 24 h following the EV injections using isoflurane and perfused with 4% paraformaldehyde. Given the timing of irradiation and euthanasia, this is an independent cohort of rats from those used in the main study (*N* = 2/group). The brains were processed for coronal sectioning using a cryostat (Leica Microsystems, Wetzlar, Germany) in the dark to preserve the dye labeling. Four serial sections (30 μm, every 10th section) were stained with DAPI and imaged across the lateral ventricle using a confocal microscope at × 40 magnification.

### Retro-orbital injections

At 72 h following the final irradiation dose (i.e. on day 8; Fig. 1), the rats were anesthetized and EVs or hibernation buffer vehicle were delivered through circulation via the retro-orbital (RO) sinus. A 31-gauge needle with a 0.5 mL syringe attached (BD Veo™ insulin syringes with BD Ultra-Fine™ 6 mm × 31G needle) was used to pierce 2–3 mm into the rat’s orbital venous sinus with the bevel on the needle facing upward at a 45° angle for injection. The control and irradiated experimental groups for both the GABAergic and glutamatergic cohorts received RO injections of 100 µl hibernation buffer (vehicle). For the GABAergic EV group, 1.5 × 10^9^ EVs in 100 µl hibernation buffer were delivered by RO injection. For the glutamatergic EV group, 1.0 × 10^9^ EVs in 100 µl hibernation buffer were delivered by RO injection. The GABAergic and glutamatergic experiments each utilize separate and independent cohorts of animals with *N* = 16/group vehicle, irradiated and irradiated + EV for each experiment.

### Behavioral cognitive testing

Five weeks after irradiation, and 2 weeks following the last RO injection, the rats underwent a battery of behavioral tasks designed to interrogate cognitive function including Novel Place Recognition (NPR), Novel Object Recognition (NOR), and Object in Place (OiP). This testing took place over the course of 2 weeks and the methods describing each of these tests have been published^8,34^. Briefly, animals were habituated to the testing room and arena for 3 days before performing the 1 day NPR task where animals were placed in an arena with two identical objects for 5 min, then returned to their housing for 1 h, and then placed back in the arena with one of the objects in its original position (“familiar”) and the other object moved to a different position (“novel”). Next, for the 1 day NOR test to evaluate hippocampal function, rats were allowed to explore two identical objects for 5 min, then removed from the arena for 5 min, then returned to the arena with one of the same (“familiar”) objects and a new (“novel”) object. The following week, OiP testing was performed, involving 2 days of habituation before the 1 day test where rats were exposed to four different objects in the arena for 5 min, then taken out of the box for 5 min. When they were subsequently returned to the arena for 5 min, the positions of two of the objects were reversed (“novel”), while the other two objects remained in place (“familiar”). For the NPR, NOR, and OiP tasks, the trials were scored for exploration time of the novel and familiar objects for each animal (*N* = 16/group) and the discrimination index was calculated from the equation: [(Novel Object/Total Exploration Time) − (Familiar/Total Exploration Time)] × 100. Following the behavioral testing the rats were randomly assigned to follow up molecular, structural or electrophysiological studies, euthanized using isoflurane anesthesia and perfused with saline + heparin alone or saline + heparin followed by 4% paraformaldehyde as appropriate.

### Extraction and ELISA for assessment of neurotrophins

Brains were immediately extracted from the skull (*N* = 4–5/group) and the hippocampus dissected from each cerebral hemisphere. Each hippocampus was weighed and transferred into 300 μL ice‐cold lysis buffer (N‐PER Neuronal Protein Extraction Reagent, Thermo Scientific) containing sodium orthovanadate (0.5 mM), phenyl‐methylsulfonyl fluoride (PMSF, 1 mM), aprotinin (10 μg/mL), and leupeptin (1 μg/mL; Santa Cruz Biotechnology, Santa Cruz, California). Tissues were sonicated individually, centrifuged at 4 °C for 15 min at 13,200 rpm, and the supernatants were collected and diluted 1:5 with Dulbecco's phosphate‐buffered saline. The supernatants were acidified to pH 2.6 then neutralized to pH 7.6 to liberate the neurotrophic factors. Following neutralization, each supernatant was diluted 1:10 prior to loading onto uncoated ELISA plates (Biolegend Nunc MaxiSorp, catalog number 423501) along with their respective BDNF or GDNF standards. The BDNF and GDNF levels were assayed using E_max_ ImmunoAssay Systems from Promega following the manufacturer’s protocol (BDNF catalog number G7611, GDNF catalog number G7621). Measurements were performed at a wavelength of 450 μm on a microplate reader (BioTek Synergy Mx) and linear regression of the standard curve was used to derive the pg of each neurotrophic factor per gram of hippocampal tissue.

### Quantification of dendritic spine density

Approximately six weeks after RO injections, animals were euthanized using isoflurane anesthesia and perfused with saline + heparin as described above. For the dendritic spine density results presented (N = 4/group), a post hoc analysis was performed where the vehicle and irradiation alone control data from both the GABAergic and glutamatergic EV treated cohorts were combined. Brains were extracted and subjected to Golgi‐Cox impregnation and staining of neurons according to the manufacturer's instructions (SuperGolgi kit, Bioenno Tech., Santa Ana, California). The brains were sectioned to 100 μm using a vibratome (Leica Biosystems; Deerpark, Illinois) and counterstained by nuclear fast red to visualize hippocampal subregions. The total dendritic spines were enumerated using the Stereoinvestigator program (v11, Microbrightfield). Briefly, serial sections (every second) through the hippocampus (AP − 3.0 mm through AP − 4.8 mm from bregma) were chosen to analyze potential differences in the number of spines between irradiated and irradiated ± EV-treated groups. Spines were counted in the molecular lacunosum (ML) layer of the dentate gyrus. Spines were counted from approximately 15–20 random but representative frames (each measuring 10 × 10 μm^2^) placed by the optical fractionator module over the ML layer. All spines were counted using the 100 × oil immersion lens with 1.5 × camera zoom. The number of frames for each section was determined using the optical fractionator (StereoInvestigator). Spine quantification from optical fractionator sampling was optimized to yield about 10–25 counted spines per frame, yielding Gundersen coefficient of error < 0.06.

### Statistical analysis

Statistical analyses were performed using one-way ANOVA to confirm overall significance along with Bonferroni’s multiple comparisons test (GraphPad Prism, v8.0, San Diego, CA). All data are presented as the mean ± SEM. All analyses considered a value of *P* ≤ 0.05 to be statistically significant.

### Supplementary Information

Supplementary Information.

## Data Availability

The data that support the findings of this study are available from the corresponding author upon reasonable request.
