# Supplementary material for: Extracellular vesicles from GABAergic but not glutamatergic neurons protect against neurological dysfunction following cranial irradiation
Source: Sci Rep. 2024 May 28;14:12274. doi: 10.1038/s41598-024-62691-y (PMC11133350; doi:10.1038/s41598-024-62691-y)
Supplement: Supplementary file 1 — Supplementary Information. [file 41598_2024_62691_MOESM1_ESM.docx]

**Supplemental Information**

**Materials and Methods**

*Behavioral cognitive testing*

Two weeks following the last RO injection, the rats underwent a battery of behavioral tasks designed to interrogate cognitive function including Novel Place Recognition (NPR), Novel Object Recognition (NOR), Object in Place (OiP) [1], and Light-Dark Box (LDB) [2]. The methods describing each of these tests have been published. The LDB task that tests anxiety in rodents, utilized an arena where one third of the box was dark and the other two thirds was a well-lit compartment (67 × 36 × 40 cm). The light and dark compartment were connected via a small opening (9 × 10 cm) that allowed the animals to freely move between the light and dark compartments. The rats were initially positioned in the center of the light compartment facing away from the opening to the dark compartment and allowed to explore freely for 10 minutes. The number of transitions between the light and dark chambers was assessed.

*Extracellular field recordings*

Hippocampal slices were prepared as previously described [3]. Following isoflurane anesthesia, rats were decapitated and the brain was quickly removed and submerged in ice-cold, oxygenated dissection medium containing (in mM): 124 NaCl, 3 KCl, 1.25 KH_2_PO_4_, 5 MgSO_4_, 0 CaCl_2_, 26 NaHCO_3_, and 10 glucose. Coronal hippocampal slices (400 µm) were prepared using a Leica vibrating tissue slicer (Model:VT1000S) before being transferred to an interface recording chamber containing preheated artificial cerebrospinal fluid (aCSF) of the following composition (in mM): 124 NaCl, 3 KCl, 1.25 KH_2_PO_4_, 1.5 MgSO_4_, 2.5 CaCl_2_, 26 NaHCO_3_, and 10 glucose and maintained at 31 ± 1°C. Slices were continuously perfused with this solution at a rate of 1.75-2 ml/min while the surface of the slices were exposed to warm, humidified 95% O_2_ / 5% CO_2_. Recordings began following at least 2 hr of incubation.

Field excitatory postsynaptic potentials (**fEPSPs**) were recorded from CA1b stratum radiatum apical dendrites using a single glass pipette filled with 2M NaCl (2-3 MΩ) in response to orthodromic stimulation (twisted nichrome wire, 65 µm diameter) of Schaffer collateral-commissural projections in CA1 stratum radiatum. Pulses were administered at 0.033 Hz using a current that elicited a 50% maximal spike-free response. After establishing a 10-20 min stable baseline, long-term potentiation (**LTP**) was induced by delivering a single episode of 5 ‘theta’ bursts, with each burst consisting of four pulses at 100 Hz and the bursts themselves separated by 200 msec (i.e., theta burst stimulation or **TBS**). The stimulation intensity was not increased during TBS.

*Statistical analysis*

LDB data are presented as the mean ± SEM (*N* = 16/group) and using one-way ANOVA with Bonferroni’s multiple comparisons test, ***P* < 0.01. LTP data in the text are presented as means ± SD, while in the figures as mean ± SEM (*N* = 4-6/group). The fEPSP slope was measured at 10–90% fall of the slope and data in figures on LTP were normalized to the last 10 min of baseline. *P* values for fEPSP slope and mean potentiation derived from one-way ANOVA with Bonferroni's multiple comparisons test. *P* values for PPF derived from two-way ANOVA. **P* < 0.05.

**Results**

*Extracellular Vesicles from GABAergic extracellular vesicles reduce anxiety following irradiation*

Animals underwent a Light-Dark Box testing paradigm to examine anxiety-like behavior, and the number of transitions between the light and dark compartment was recorded. Significant overall group effects were detected (F_(2, 45)_ = 6.466; *P* = 0.0034), and the control animals exhibited a significantly higher (F_(2, 45)_ = 6.466; *P* = 0.0027) number of transitions (mean number of transitions = 3.750) compared to irradiated animals (mean number of transitions = 1.188; **Supp. Fig. 1**). The GABAergic EV-treated group likewise had a higher number of transitions (mean number of transitions = 2.125) relative to the irradiated group, but this difference was not statistically significant (F_(2, 45)_ = 6.466; *P* = 0.6005, Fig. 5). Thus, treatment with GABAergic EVs does not significantly ameliorate irradiation-induced anxiety-like behavior.

*Hippocampal and cortical synaptic plasticity is perturbed by irradiation*

We assessed LTP after the conclusion of behavior studies in hippocampal brain slices prepared from cranially irradiated and concurrent control animals **(Supp. Fig. 2)**. The delivery of five theta burst stimulations to control hippocampal slices caused the predicted rapid increase in the slope of the field fEPSPs followed by decay over ten minutes to a stable plateau above pre-TBS baseline in all groups (159 ±12%, 95% CI; **Supp.** **Fig. 2A**). The level of potentiation in fEPFS slope maintained 50-60 minutes post-TBS was reduced significantly in the hippocampus of the irradiated animals (*P* = 0.0355) but not in the irradiated mice that received EVs (*P* = 0.319) relative to controls (**Supp. Fig. 2B).** Measures of baseline synaptic transmission were found to be unaltered in the irradiated or irradiated GABAergic EV group (slopes of io curves; F (2,13) = 0.84, *P* = 0.45) (**Supp Fig. 2C**). Accordingly, there were no significant changes in presynaptic plasticity of transmitter release (2-way ANOVA; F (4,26) = 0.61, *P* =0 .66) as measured in a paired pulse facilitation assay (**Supp Fig. 2D)**. Together these results suggest that impaired LTP found after irradiation could not be significantly restored by GABAergic EV treatments.


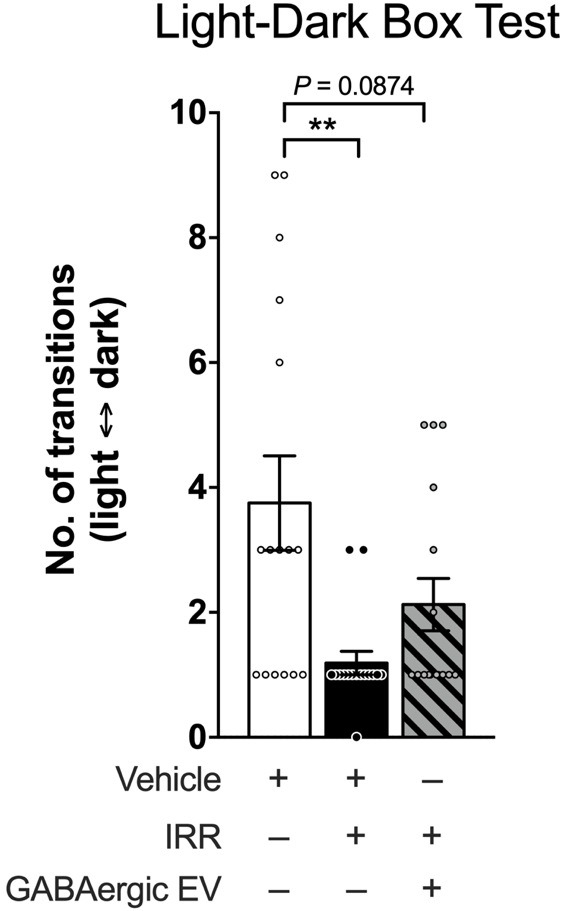


**Supplemental Figure 1:** **Treatment with GABAergic extracellular vesicles (EVs) does not reduce radiation induced anxiety behavior**. The mean number of transitions was higher in the animals treated with GABAergic EVs as compared to the irradiated group, though not to a statistically significant. Data are presented as the mean ± SEM (*N* = 16/group). ***P* < 0.01, one-way ANOVA with Bonferroni’s multiple comparisons test.


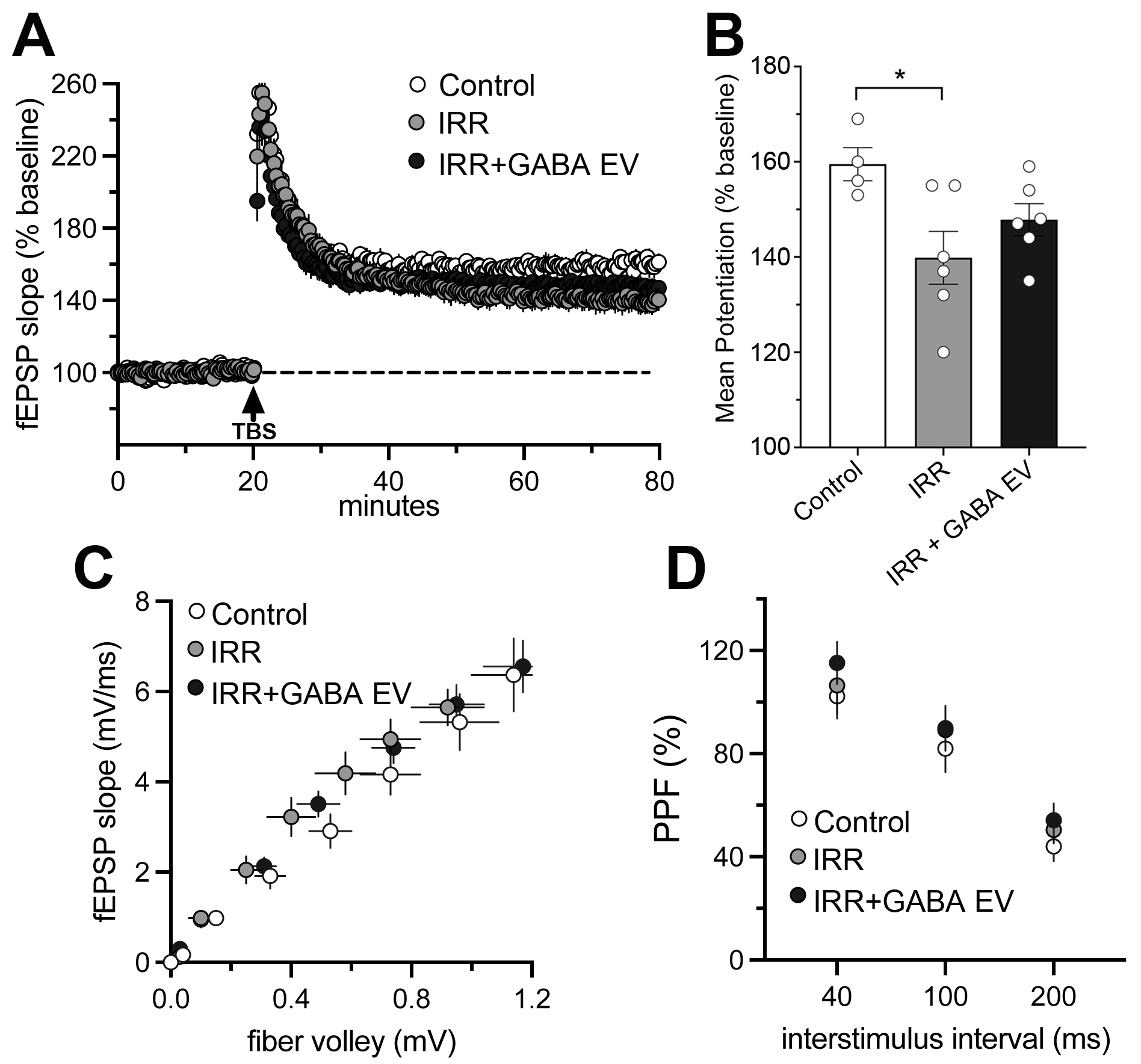


**Supplemental Figure 2:** **Theta burst-induced LTP is notably reduced in slices from irradiated animals, but not in those treated with GABAergic extracellular vesicles (EVs)**. **A, B).** Theta burst-induced LTP is impaired in slices from irradiated animals as compared to slices from irradiated and GABAergic EV treated group or the control group. **C).** Short-term potentiation, including input/output curve and **D)** and paired-pulse facilitation were not measurably different among all three groups. Data are presented as mean ± SEM (*N* = 4-6 animals/group). *P* values for fEPSP slope and mean potentiation derived from one-way ANOVA with Bonferroni's multiple comparisons test. *P* values for PPF derived from two-way ANOVA. **P* < 0.05.

**Supplemental References**

1 Acharya MM, Christie LA, Lan ML et al. Human neural stem cell transplantation ameliorates radiation-induced cognitive dysfunction [in eng]. Cancer Res 2011;71(14):4834-4845.

2 Dey D, Parihar VK, Szabo GG et al. Neurological Impairments in Mice Subjected to Irradiation and Chemotherapy. Radiat Res 2020;193(5):407-424.

3 Limoli CL, Kramar EA, Almeida A et al. The sparing effect of FLASH-RT on synaptic plasticity is maintained in mice with standard fractionation. Radiother Oncol 2023;186:109767.
